# Thiamine Deficiency Is Common and Underrecognized in Emergency Department Oncology Patients

**DOI:** 10.3390/jcm14010257

**Published:** 2025-01-04

**Authors:** Deepika Boopathy, Daniel Grahf, Jacob Ross, Kegham Hawatian, Jo-Ann Rammal, Katherine Alaimo, Joseph B. Miller

**Affiliations:** 1 Department of Public Health Sciences, Henry Ford Health, Detroit, MI 48202, USA; 2 Departments of Emergency Medicine, Advocate Christ Hospital, Chicago, IL 60453, USA; 3 Departments of Internal Medicine, Henry Ford Hospital, Detroit, MI 48202, USA; 4 Department of Emergency Medicine, Henry Ford Health, Detroit, MI 48202, USA; 5 Department of Food Science and Human Nutrition, Michigan State University, East Lansing, MI 48824, USA

**Keywords:** oncology, emergency medicine, cancer, thiamine deficiency, Wernicke’s encephalopathy, micronutrients

## Abstract

**Background**: Wernicke’s encephalopathy can occur in oncology patients independent of alcohol use, likely resulting from poor dietary thiamine intake. High metabolic demands, such as those in acute illnesses seen in the emergency department (ED), can exacerbate thiamine deficiency. In this study, our objective was to assess the incidence of thiamine deficiency in ED oncology patients, which could lead to Wernicke’s encephalopathy or other thiamine deficiency disorders if left untreated. **Methods**: This was a single-center prospective cohort study. We included patients with acute illness and a history of active cancer management in the ED of a large, urban hospital. We also included age and sex-matched control patients with no history of cancer who sought ED care. We excluded patients with a history of alcohol use or parenteral thiamine administration before enrollment. We recorded whole blood thiamine levels to measure total body thiamine stores and collected data on clinical variables, thiamine treatment, and adverse events. **Results**: In total, 87 oncology and 71 control patients were included in the study. The mean age was 62.1 ± 13.7 and 58.9 ± 12.6 years, respectively, and 48% of oncology vs. 55% of control participants were female. The most common cancers represented were colon (23%), lung (25%), prostate (10%), and breast (9%). Thiamine deficiency was significantly higher in ED oncology patients (25, 28.7%) compared to controls (6, 8.5%), odds ratio 4.4 (95% CI 1.7–11.4). None of the oncology patients with deficiency received thiamine treatment in the ED. **Conclusions**: Our findings suggest that thiamine deficiency is prevalent in acutely ill oncology patients, yet rarely treated in the ED.

## 1. Introduction

Thiamine is an essential water-soluble vitamin that requires continuous dietary intake to support carbohydrate metabolism, brain function, and peripheral nerve myelination. Recent research has shown an unexpectedly high prevalence of thiamine deficiency in critically ill populations other than those with alcohol dependence [1], and among acutely ill patients with advanced age and leukopenia presenting to the ED [2]. A common link between these different populations is an acute increase in metabolic demand. Poor dietary intake, acute illness, and high metabolic demand may rapidly deplete thiamine stores. Thiamine deficiency results in Wernicke’s encephalopathy, is associated with poor outcomes in critically ill patients, and is correlated with other derangements in patients with comorbidities [3,4,5]. Thus, its impact on acutely ill patients presenting to the ED can aid with prognostication, disposition, and possibly with direct medical treatment in the ED.

The literature abounds with reports of Wenicke’s encephalopathy in oncology patients. Still, prospective assessments of thiamine deficiency in this population are lacking in the setting of acute illness, such as in the emergency department (ED) [6,7]. Even in the absence of alcohol dependence, patients with active cancer and acute illness have a substantial risk for poor dietary intake and high metabolic demands.

Thiamine deficiency is a specific possible consequence of said poor dietary intake and increased metabolic demand in cancer patients, as there is growing evidence that tumors consume thiamine at a greater rate as compared to normal body tissue [8,9]. So, in the light of oncologic patients having several prediposing factors towards thiamine deficiency, and the relatively increased prevalence of Wernicke’s encephalopathy in this patient patent population, we sought to test the hypothesis that thiamine deficiency occurs frequently in the acutely ill ED oncology patient population. Our overarching goal is to uncover how clinicians can best assess oncology patients for thiamine deficiency when they present to the ED or hospital with acute illness.

Additionally, we conducted a literature search concerning the biochemical dynamics of thiamine in cancer and cancer patients to better understand our analysis and findings.

## 2. Objective

The primary goal of this study was to assess the incidence of thiamine deficiency in acutely ill oncology patients compared to matched controls. Secondary outcomes included assessment of death and the diagnoses of delirium or Wernicke’s encephalopathy.

## 3. Materials and Methods

### 3.1. Design, Setting, and Population

This was a prospective cohort study of adult patients presenting to an urban tertiary care center ED for any complaint between July 2017 and October 2018. We identified adults (age 18 years and older) receiving current treatment for any known malignancy in the ED. We identified controls as ED patients with matched age and sex but no known malignancy. For each oncology patient enrolled, we limited the next control enrollment to the same biological sex and similar age (within five years). For oncology and matched control patients, we excluded those with a self-reported history of alcohol use or those who received empiric thiamine before enrollment. Enrollment occurred as a convenience sample based on research team availability. The study was approved by the health system’s Institutional Review Board, and a waiver of consent was granted for patient enrollment.

### 3.2. Study Procedures

For each patient, we obtained whole blood thiamine in conjunction with their blood draw for standard care labs in the ED. We used light-protected tubes for blood collection and immediately froze whole blood below −70 °F for shipment to the reference lab. The whole blood level was measured using high-performance liquid chromatography (Warde Laboratories, Ann Arbor, MI, USA). We defined thiamine deficiency as a level below the lowest limit of the reference range (38–122 µg/L). Study team members also collected patients’ demographics, clinical characteristics, and standard care laboratory results. We also assessed mortality within 60 days and the presence of delirium or Wernicke’s encephalopathy, as determined by diagnoses made by the treating clinicians. Study team members made thiamine levels available to treating physicians when they became available, usually within five days of the ED visit.

### 3.3. Statistical Analysis

We performed descriptive statistics and univariate comparisons of cases and controls. We used a Student’s *t*-test for continuous variables; we used chi-square or Fischer’s exact test where indicated for binary variables. The primary outcome analysis used logistic regression to assess the main outcome of thiamine deficiency. We further performed multivariable logistic regression to account for age, sex, and hypoalbuminemia. Due to the exploratory nature of this study, we did not conduct a formal sample size estimate and power analysis. We performed all analyses with SAS 9.4 (Cary, NC, USA) and reported unadjusted odds ratios (OR) or adjusted OR (aOR) with 95% confidence intervals (CI).

## 4. Results

**Figure 1:**
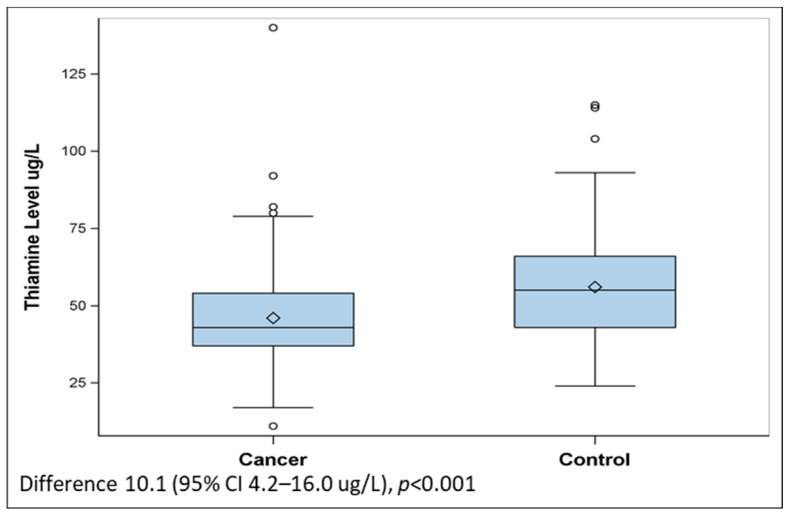
Comparison of whole blood thiamine levels in patients with active cancer compared to matched controls.

We enrolled 87 oncology patients and 71 control patients. The imbalance in enrollment occurred due to funding constraints to conclude the enrollment of control patients. Table 1 demonstrates the demographic and clinical characteristics. Among the oncology cohort, the most common malignancies were colon, lung, prostate, and breast. Oncology patients had higher rates of anemia and hypoalbuminemia. The mean thiamine level among oncology patients was lower than controls (difference 10.1, 95% CI 4.2–16.0 ug/L, Figure 1).

In the oncology cohort, 25 (28.7%) patients had thiamine deficiency compared to 6 (8.5%) among control patients (OR 3.02, 95% CI 1.26–7.27, *p* = 0.014). Table 2 demonstrates factors associated with thiamine deficiency. Adjusting for age, sex, and hypoalbuminemia, oncology patients continued to have significantly higher odds of thiamine deficiency compared to control patients (aOR 3.09, 95% CI 1.28–7.47, *p* = 0.012). No patient with thiamine deficiency received empiric thiamine replacement in the ED.

The control population had no deaths within 60 days. However, among oncology patients, 14 (16.1%) deaths occurred. This death rate was higher among thiamine-deficient patients (20.8%) than among non-deficient patients (14.3%), although not statistically significant (OR 1.58, 95% CI 0.47–5.30, *p* = 0.460). Among the oncology cohort, delirium was also more common in thiamine-deficient patients (20.8%) compared to non-deficient patients (11.3%), a difference which was not statistically significant (OR 2.07, 95% CI 0.59–7.29, *p* = 0.259). No patients had a formal diagnosis of Wernicke’s encephalopathy.

## 5. Discussion

### 5.1. Clinical Elements

In this small study of acutely ill oncology patients and matched controls, oncology patients in the ED had significantly higher rates of thiamine deficiency (28.7%). This indicates a population that may benefit from preventative thiamine supplementation and parenteral replacement in the ED and hospital setting. As has been described in critically ill patient populations, we suspect the combination of high metabolic demand and poor nutrition contributes to these findings [1,10]. Preclinical studies have also demonstrated that tumor growth may deplete tissue thiamine stores while cancer cells maintain constant thiamine levels [11,12].

The high 60-day mortality rate among oncology patients in this study indicates that a substantial portion of patients were near the end of their lives and may have had notably diminished nutritional reserve. Although the treating teams made no diagnoses of Wernicke’s encephalopathy, our data suggest higher rates of delirium in thiamine-deficient patients.

### 5.2. Thiamine Biochemistry in Malignancy

Conflicting conclusions are drawn from studies examining the molecular behavior of thiamine in cancer.

Thiamine carriers THTR1 and THTR2 (SLC19A2 and SLC19A3) mediate thiamine absorption. Reduced expression of THTR2 (SLC19A3) was found in malignant breast [13], gastric [14], and colon cancer as compared to physiologic expression in normal tissue. While there is a decreased expression of one known thiamine transporter, this may or may not impact overall thiamine levels, as the compensatory role of the remaining transporter is unknown. How this plays into thiamine serum levels and the amount of thiamine delivery to neural tissue is still under study.

The specific metabolism of thiamine is also changed in malignant tissue. One study displayed constant thiamine levels in tumorigenesis tissue, coupled with a constant thiamine level decline in healthy host liver tissue [15]. A similar conclusion of increased thiamine levels in cancerous tissue was drawn in the case of pathological specimens of excised or autopsy-sourced colon adenocarcinoma tissue. These findings suggest an increased usage of thiamine by malignant cells, leading to an equivalent reduction in thiamine levels both in the serum and in healthy tissue, including organ systems where thiamine deficiency is most noticeable, such as the central nervous system. However, there is limited evidence to explain the biochemical pathway behind this occurrence.

An additional mechanism for thiamine deficiency is made possible by the side effects of chemotherapeutic drugs, such as 5-fluorouracil and ifosfamide. Both drugs have shown association with a thiamine deficient state [16,17]. The extent to which chemotherapeutic agents affect thiamine homeostasis independent of the underlying malignant process is poorly defined, and warrants further investigation.

The clinical relevance of this thiamine dysregulation is still contentious, but neurological dysfunction that could be compared to Wernicke’s encephalopathy has been observed in patients with abnormal thiamine levels [17,18,19,20]. However, as with the results discussed in this section, the causality link is unclear and requires a closer look.

Since a sizeable body of evidence shows an increased uptake of thiamine by malignant cells, a logical next question is whether thiamine-deficient patients with malignancy should be supplemented with exogenous thiamine. The cellular influence of thiamine on tumorigenesis and tumor aggression has been documented in a number of accounts.

A dose–response examination of thiamine level influence on tumor behavior yielded a graded response. The effect of low-to-moderate levels of thiamine supplementation to the tumor microenvironment, levels up to 37.5 times the recommended daily intake (RDI), yielded a significant stimulatory effect on tumor activity, increasing proliferation [12]. The high RDI level should not be seen as unrealistic to achieve in actual patients, as there is a strong suggestion that tumor cells are able to achieve higher thiamine levels as compared to regular body tissue, as we have detailed prior. At 75 times the RDI, the dose–response relationship between thiamine supplementation and increased tumor proliferation was lost, revealing a possible but ill-defined thiamine saturation point. At 2500 times RDI levels, tumor proliferation was decreased as compared to no supplementation.

Summarily, in this one study [12], thiamine levels were shown to increase tumor proliferation up to a certain point (37.5 times RDI), after which the dose–response is lost (75 RDI and upwards) and then replaced by a slight suppressive effect at thiamine megadose (2500 RDI). The clinical relevance of this information remains to be elucidated.

Thiaminase was shown to reduce ATP levels and hereby possibly impeding malignant cell activity in cancer tissue. Tumor growth was reduced and survival was extended in a xenograft leukemia model upon thiaminase supplementation [21]. Additionally, oxy-thiamine, a thiamine anti-coenzyme, was shown to decrease malignant cell growth in both in vitro and in vivo tumor cell models [22,23,24].

This body of evidence points towards increased thiamine levels increasing tumor activity, growth, and aggression. The specific influence in human patients remains to be studied, as the specific biochemistry, levels, and dose–response relationships between patient thiamine supplementation and clinically relevant tumor activity is unknown. However, the findings warrant a cautionary note against supplementing patients with active malignancy with thiamine, as it very well could be a promoter of tumor health and activity.

The overarching theme for thiamine metabolism in cancer patients is a downregulation of the transporters that enable thiamine transfer into the CNS, along with an increased consumption of thiamine by malignant tissue, which appears to be conducive to tumor survival, proliferation, and aggression. Thiamine is shunted away from the CNS and to tumor tissue, increasing the risk of Wernicke’s while also aiding tumorigenesis and malignance.

This presents a conundrum, as thiamine can safely be assumed to be metabolized more heavily by cancerous tissue, and thereby consumed more heavily in patients with malignancy, predisposing them to thiamine deficiency. A balance between the negative effect of thiamine deficiency and the dangers of encouraging tumor growth by supplementing thiamine—and possibly increasing the thiamine metabolism and deepening the problem—should be described for a clinical recommendation to be given.

In other words, examining the biomechanics of thiamine supplementation is needed. Does the supplied thiamine feed the tumor, or help stave off Wernicke’s and neurological dysfunction? To what extent is thiamine restriction for tumor suppression worth the risks of thiamine deficiency?

An additional possible point of exploration could be thiamine supplementation directly to the deficient tissue while avoiding supplying the tumor. Since thiamine is lipophilic and does not cross the blood–brain barrier, intrathecal delivery of thiamine, to supply neurological tissue without providing tumors with metabolic strength, could be worth exploring.

Since malignancy appears to downregulate serum thiamine transporters, which slows down the process by which thiamine is delivered to neural tissue, then one can theorize that direct CNS delivery of thiamine through the intrathecal route could be viable. Since thiamine transporter expression is reduced, thiamine could be restricted to the CNS with little diffusion back into the blood, and, consequently, to tumor tissue.

This could present a possible solution for supplying thiamine deficient oncologic patients at risk of developing Wernicke’s encephalopathy with thiamine with reduced or no risk of increasing tumor activity. However, this is a theoretical suggestion with no substantiation, and its viability will be validated or disproved by a deeper understanding of the biochemical behavior of thiamine transporters within an organ system with malignancy.

Given our current understanding of the biochemical premises of thiamine levels in oncologic patients, thiamine level could perhaps be used as a nutritional reserve surrogate marker, a measure of tumor burden on the nutritional status of the patient, or even a prognostication variable for acutely ill oncology patients presenting to the ED. Further investigation is needed to substantiate these possibilities.

### 5.3. Limitations

This study had several limitations. We did not perform standardized clinical or imaging assessments for Wernicke’s encephalopathy or formal nutritional evaluations to gauge the degree of malnourishment among participants. The sample was also too small to adjust for many additional factors, such as cancer subtype, presence of metastatic disease, and additional comorbid conditions or medications. Conditions such as heart failure and diuretic use are associated with thiamine deficiency [25,26,27]. Other less common conditions that influence thiamine levels require a larger sample size to be assessed accurately.

While limited in scope, our study adds to a growing body of evidence that deficiencies in thiamine, and likely other micronutrients, are common in patients with active cancer and acute illness [2,6,7,28]. Further investigation is needed to validate the presence of thiamine deficiency more commonly in oncology patients in the ED. Additionally, studies with larger sample sizes would be able to verify whether the clinically significant outcomes, mainly mortality, delirium, and Wernicke’s encephalopathy, are more common in oncology patients diagnosed with thiamine deficiency in the ED.

Given the absence of point-of-care laboratory evaluation of thiamine deficiency, and the time window needed for thiamine levels to be apparent upon laboratory testing, clinical decision support tools or novel point-of-care devices are needed to aid ED and hospital clinicians in determining patients most likely to benefit from thiamine replacement. Furthermore, additional research is needed to determine the burden of micronutrient deficiencies, particularly in regard to water-soluble vitamins, in the setting of acute illness and underlying malignancy.

## 6. Conclusions

Our findings suggest that thiamine deficiency occurs commonly in acutely ill oncology patients, yet is rarely treated in the ED. Further studies that comprehensively assess all possible clinical characteristics that have an influence on thiamine level, such as diuretic intake, gastrointestinal diseases, thyrotoxicosis, and inflammatory diseases, in a larger patient population, are needed to validate or rectify our preliminary conclusion of thiamine deficiency being more common in acutely ill oncology patients.

## Figures and Tables

**Table 1:** Demographic and clinical characteristics of oncology and control patients.

Characteristics	Oncology Patients	Control Patients
Total Patients	87	71
Age in Years—mean ± SD	62.1 ± 13.7	58.9 ± 12.6
Female Sex—no. (%)	42 (48.3)	39 (54.9)
Race—no. (%)		
Black	55 (63.2)	57 (80.3)
White	25 (28.7)	8 (11.3)
Other	7 (8.0)	6 (8.5)
Comorbidities—no. (%)		
Coronary artery disease	7 (8.1)	3 (4.2)
Congestive heart failure	8 (9.2)	3 (4.2)
Stroke	8 (9.2)	6 (8.5)
Anemia	16 (18.4)	5 (7.0)
Chronic kidney disease	13 (14.9)	4 (5.6)
Hypertension	57 (65.5)	44 (62.0)
Diabetes Mellitus	20 (23.0)	27 (38.0)
Body Mass Index—mean kg/m^2^ ± SD	26.8 ± 6.7	29.4 ± 8.0
Laboratory values—no. (%)		
Creatinine > 1.5 mg/dl	13 (14.9)	7 (9.9)
Albumin < 3.5 mg/dL	25 (28.7)	6 (8.5)
Hemoglobin < 10 mg/dL	31 (35.6)	11 (15.5)
Low thiamine (<38 µg/L)	25 (28.7)	6 (8.5)
Thiamine—mean, µg/L ± SD	46.0 ± 18.9	56.1 ± 19.3
Cancer Type—no. (%)		
Colon and Gastrointestinal	20 (23.0)	--
Lung	23 (25.4)	--
Prostate	9 (10.3)	--
Genitourinary	6 (6.9)	--
Head and Neck	3 (3.4)	--
Gynecological	7 (8.0)	--
Breast	8 (9.2)	--
Hematologic	6 (6.9)	--
Other	3 (3.4)	--

**Table 2:** Unadjusted and adjusted analysis of patient characteristics associated with thiamine deficiency.

	Unadjusted Analysis *		Adjusted Analysis	
	Odds Ratio (95% CI)	*p*-Value	Odds Ratio (95% CI)	*p*-Value
Female Sex	1.78 (0.80–3.94)	0.158		
Age	1.00 (0.97–1.03)	0.868		
Cancer	3.02 (CI 1.26–7.27)	0.014	3.09 (1.28–7.47)	0.012
Albumin < 3.4 g/dL	1.25 (0.56–2.78)	0.584		

* Categorical variables included in logistic regression model. Adjusted analysis displays only results for retained variables with *p* < 0.05.

## Data Availability

Datasets used and/or analyzed during the current study are available from the corresponding author on reasonable request.
